# Dietary Intervention With Lutein, Docosahexaenoic Acid, and Choline During Early Development Improves Recognition Memory in Young Adult Females in an Experimental Model

**DOI:** 10.1002/mnfr.70589

**Published:** 2026-08-27

**Authors:** Leon Saschin, Gregor Fink, Maria Wohlfarth, Jonas Hauser, Jian Yan, Dirk Wiedermann, Mathias Hoehn, Jochen Wilhelm, Lea Quell, Elisabeth Sous, Jenny Voggel, Jörg Dötsch, Eva Nüsken, Kai‐Dietrich Nüsken

**Affiliations:** ^1^ Department of Pediatric and Adolescent Medicine, Faculty of Medicine and University Hospital Cologne University of Cologne Cologne Germany; ^2^ NPTC Nutrition Konolfingen Switzerland; ^3^ Nestlé Research Société des Produits Nestlé S.A. Lausanne Switzerland; ^4^ Max Planck Institute for Metabolism Research Cologne Germany; ^5^ Member of the German Center for Lung Research (DZL) Institute for Lung Health (ILH) University of Giessen and Marburg Lung Center (UGMLC) and Cardiopulmonary Institute (CPI) Giessen Germany

**Keywords:** antioxidants, developmental programming, lutein, memory, nutrition

## Abstract

The perinatal period and adolescence are critical for brain development, with nutrition being a crucial factor. Dietary intake of lutein, docosahexaenoic acid (DHA), and choline is positively associated with early neurodevelopment and cognitive function, but interactions remain unclear. This study systematically evaluates lutein, DHA, and choline to elucidate their specific and combined effects on neurodevelopment.

To ensure fully controllable conditions, we performed nutritional interventions on a rat model during a critical developmental phase (days 14–56 after birth). After completion of the intervention, no relevant alterations in the open field test or the elevated zero maze were detected. However, both the combination of lutein, DHA, and choline and lutein monotherapy led to an increase in the recognition index for new objects in female rats. No relevant effects on blood and metabolic parameters were observed in any group. In vivo MRI analyses ruled out differences in brain morphology. Our results provide evidence that lutein supplementation during brain development has positive effects on recognition memory in female rats, underscoring the need for sex‐specific research on nutritional strategies. Lutein supplementation, particularly in combination with DHA and choline, to enhance neurological development potential should be investigated in further translational studies.

## Graphical abstract

Our study investigates the effects of perinatal supplementation with lutein, DHA, and choline in a controlled rat model. The results show: (1) strictly sex‐specific effects exclusively observed in female rats, (2) lutein‐dependent and synergistic effects, (3) enhancement of long‐term recognition memory, and (4) no effects on locomotor activity, anxiety‐like behavior, clinical blood parameters, or in vivo brain MRI.

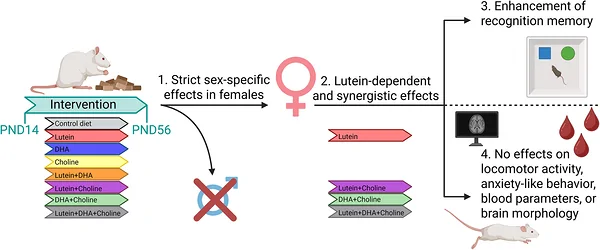

**Abbreviations**
CI: confidence interval
DHA: docosahexaenoic acid
DTI: diffusion tensor imaging
FA: fractional anisotropy
GD: gestational day
MRI: magnetic resonance imaging
MSME: multi‐slice multi‐echo
NOR: novel object recognition
PND: postnatal day
PUFAs: polyunsaturated fatty acids
ROS: reactive oxygen species

## 1 Introduction

Favorable conditions during an organism's development can have lifelong positive effects on organ function and overall health. The concept of early life programming originated in research on the development of metabolic diseases, but has recently been extended to immune function and brain development [1, 2, 3]. In mammals, including both humans and rats, the perinatal period and adolescence are crucial stages of brain development, characterized by increased neuroplasticity, synaptic plasticity, and glial proliferation. During these phases, the brain is particularly sensitive to environmental factors and the composition of the diet [4, 5].

New findings underscore the crucial role of nutrition in functional outcomes, with early nutritional components such as antioxidants or lipids having a lasting effect on cognitive and metabolic health [1, 6, 7, 8, 9]. Due to its high metabolic demands, elevated tissue concentrations of polyunsaturated fatty acids (PUFAs), and relatively low antioxidant capacity, the developing brain is particularly vulnerable to oxidative stress [10, 11]. Consequently, early dietary interventions may not only support optimal neurodevelopment but also mitigate oxidative damage. Key dietary compounds such as lutein (an antioxidant), docosahexaenoic acid (DHA), and choline have demonstrated significant benefits for cognition and neural maturation. Preclinical studies suggest that early‐life choline supplementation enhances neurocognitive function, including auditory processing acuity and temporal signal discrimination, compared to standard diet [12]. In humans, a prospective cohort study revealed synergistic effects of DHA and choline on recognition memory [13], with similar associations observed for lutein and choline. Another cohort study revealed better verbal intelligence and behavior regulation ability in mid‐childhood, when the maternal lutein and zeaxanthin intake during pregnancy was higher [14, 15]. Notably, a recent maternal cohort study further supports the combined impact of prenatal lutein, DHA, and choline intake on fetal neurodevelopment [16], highlighting the potential for multi‐dietary‐factor‐strategies to optimize early brain health. Several factors contribute to the fact that the combined administration, in particular, offers measurable benefits. First, taking DHA and choline together increases their availability in the brain, as it enables the synthesis of larger amounts of DHA‐containing lysophosphatidylcholines (PCs), which significantly facilitates their passage through the blood‐brain barrier [17]. This improves neurocognitive function [18]. Second, it has been shown that DHA supplementation significantly increases cellular choline uptake, thereby stimulating choline acetyltransferase activity and promoting the growth of neural cells [19, 20]. Furthermore, DHA and choline interact with each other as components of cell membranes and affect the survival and death of neurons [21, 22, 23]. Third, because of its unsaturated chemical structure, DHA is highly susceptible to oxidative damage. This susceptibility can be reduced by taking additional lutein, which acts as a powerful antioxidant, as well as choline [24, 25]. A triple‐action dietary supplement can therefore enhance the individual effects of each nutrient.

The aim of this study was to systematically evaluate the effect of lutein, DHA, and choline (both individually and in all possible combinations) under fully controlled conditions in order to gain new insights into their individual and additive effects. We provide a comprehensive and multidimensional assessment of metabolic and blood parameters, brain morphology, and neurocognitive behavior in response to nutritional interventions. The behavioral analysis was designed to test three predefined and interdependent hypotheses: (1) whether combined lutein, DHA, and choline supplementation improves novel object recognition (NOR), (2) whether lutein is necessary for this effect, and (3) whether lutein alone is sufficient to enhance recognition memory. The rigorous comparative approach enables evidence‐based conclusions to be drawn about the most effective nutritional strategies for optimizing neurological development and metabolism.

## 2 Experimental Section

### 2.1 Ethical Statement

All animal procedures were conducted in strict compliance with German federal regulations and approved by the relevant governmental authorities (License ID: 84‐02.04.2016.A129).

### 2.2 Animal Housing

Time‐mated Wistar rat dams (Janvier Labs, France) were individually housed under controlled conditions (22°C, 12/12 h light/dark cycle) with ad libitum access to water and a standard control diet (Altromin C1000: 67% carbohydrates, 20% protein, 13% fat; 3506 kcal/kg). From gestational day (GD) 14, dams were acclimatized to the facility.

Pups were delivered spontaneously within 12 h on GD23 [designated postnatal day (PND) 1]. To standardize conditions, only litters with 9–15 pups (mean: 12) were included. On PND2, litter size and sex were recorded, and litters were adjusted to 8 pups per dam (four males, four females) via cross‐fostering. Pups were randomly selected from four different litters per foster dam (one male + one female per litter).

From PND14 to PND56, offspring were allocated to 8 dietary groups [(2 foster dams/group × 2 experimental periods; total *n* = 16 dams, 64 males, 64 females); specific dosage information in following section].

**FIGURE 1:**
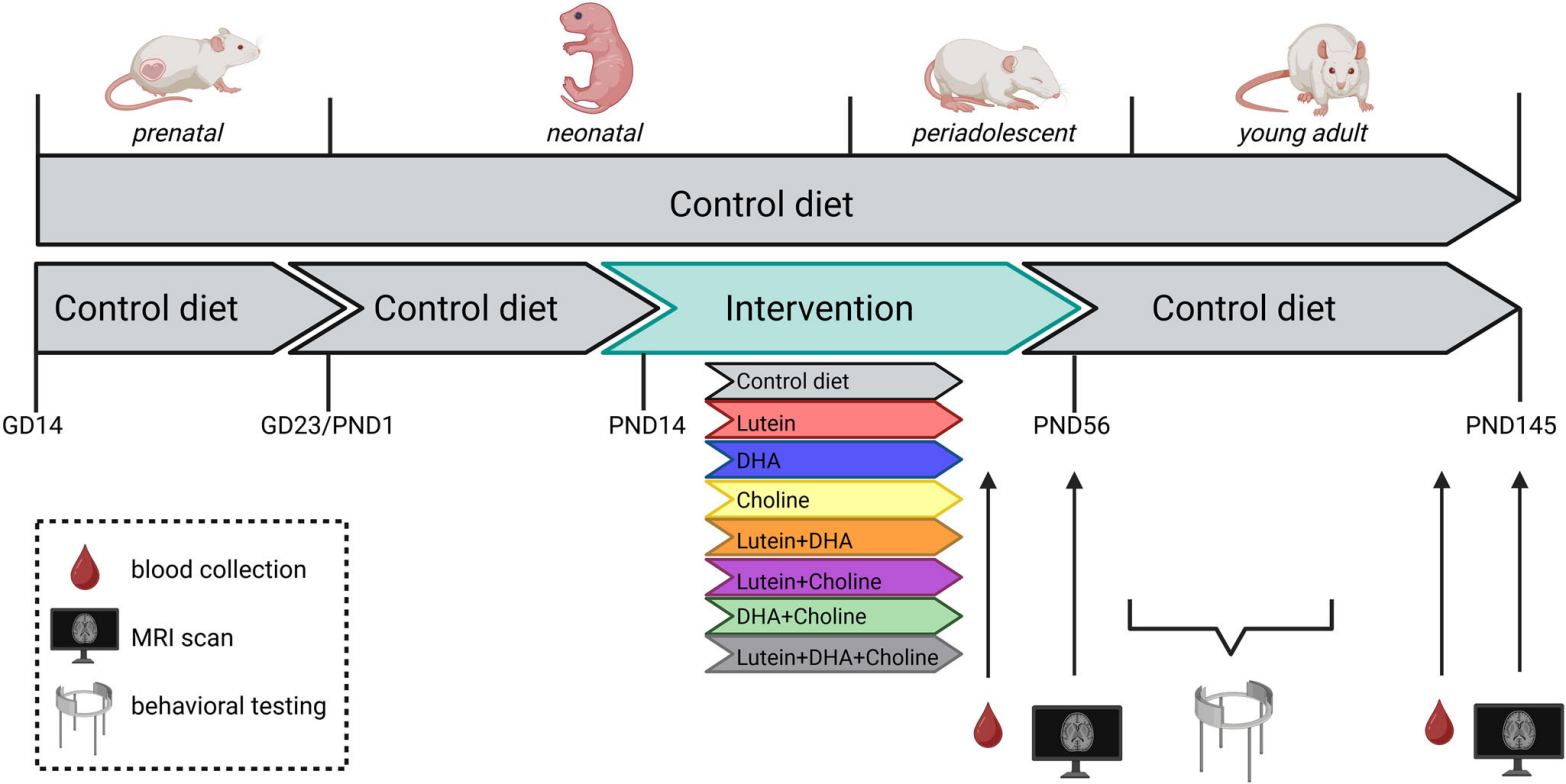
Schematic of the experimental design and dietary intervention timeline. From gestation day (GD) 1, dams were fed a control diet. Pups were born on GD23 (designated PND1). At PND14, pups were weaned and randomly assigned to one of eight experimental groups receiving a control diet or a control diet supplemented with Lutein, DHA, Choline, or their combinations until PND56. Blood collection for metabolic and clinical analysis was performed at PND33‐35/PND120‐133, magnetic resonance imaging (MRI) for neurostructural assessment was conducted at PND53‐69/PND137‐151, and behavioral testing took place from PND64‐87. GD, gestational day; PND, postnatal day; DHA, docosahexaenoic acid; MRI, magnetic resonance imaging.

After weaning (PND21), offspring were group‐housed (*n* = 2–4) with environmental enrichment. Blood sampling was performed on PND33‐35 and PND146‐148 after a 6‐h fasting period (7 a.m. to 1 p.m.). Briefly, 1.5 mL of blood was collected via retro‐orbital puncture under isoflurane anesthesia, with the procedure completed within 30 s to minimize stress. From PND36 onwards, animals (*n* = 16 per group, balanced by sex) were transferred to “metabolic” cages for 24‐h intake‐ and output monitoring of water and urine as well as food and feces. This protocol was repeated on PND120‐133 prior to the study endpoint to assess longitudinal metabolic changes. For terminal tissue collection (PND152‐160), animals were deeply anesthetized with isoflurane followed by an intraperitoneal injection of pentobarbital (Narcoren, 16 g/100 mL; dose, 5 mL/kg bodyweight). Upon absence of pain reflexes, transcardial perfusion with PBS was performed. The whole brain was rapidly extracted, weighed, further processed, and stored. Concurrently, other organs and tissues (liver, skeletal muscle, retroperitoneal, subcutaneous and mesenteric adipose depots, eyes, kidneys, and epididymal fat tissue) were harvested, weighed, partially further processed, and all stored. For an overview of the experimental design, please see Figure 1.

### 2.3 Dosage Information/Dosage Regimen

This study aimed to evaluate the impact of dietary composition rather than absolute nutrient intake. Diets were based on published animal studies and formulated such that standard ad libitum consumption would ensure sufficient exposure to the supplemented nutrients (lutein, DHA, choline). To minimize handling stress, food intake was quantified once on PND40. From PND14 to PND56, offspring were assigned to one of eight feeding groups based on Altromin C1000 (composition provided in Table S5) and enriched with the following supplements to cover the critical period for neurological development:

1. Control diet  (Altromin C1000, no supplementation)
2. Lutein  (Altromin C1000 + 100 mg lutein/kg food)
3. DHA  (Altromin C1000 + 5 g DHA/kg food)
4. Choline  (Altromin C1000 + 5 g choline chloride/kg food)
5. Lutein + DHA
6. Lutein + Choline
7. DHA + Choline
8. Lutein + DHA + Choline

Using the measured average daily food intake (females: 28 g; males: 35 g) and mean body weight on PND42 (females: 181 g; males: 228 g), the estimated daily nutrient dosages were as follows:

Lutein: ∼15 mg lutein/kg body weight/day
DHA: ∼750 mg DHA/kg body weight/day
Choline: ∼750 mg choline/kg body weight/day

The specific nutrient concentrations were selected based on scientific literature and recognized publications, with particular attention paid to safety [12, 26, 27]. The doses were selected to ensure a high level of exposure that is also well tolerated. Under standardized experimental conditions, nutrient supply was maintained throughout the study to ensure a consistently adequate intake of the nutrients. Inter‐species differences in body size, body weight, and metabolic rate were accounted for using a standardized allometric scaling method that converts the administered doses into human equivalent doses, thereby supporting the relevance and generalizability of the results. In terms of timing, the dietary intervention spanned days 14 through 56 after birth, which corresponds to late infancy and childhood in humans—a critical period for neurological development.

### 2.4 Behavioral Tests

#### 2.4.1 Elevated Zero Maze (PND 64–66)

Anxiety‐related behavior was evaluated using the elevated zero maze (diameter: 100 cm), which was performed on PNDs 64–66; that is, 8–10 days after end of the dietary intervention. The zero maze consisted of two opposing open quadrants and two enclosed quadrants (wall height: 15 cm). Animals were placed in a closed quadrant under 60 lux illumination and observed for 6 min. Primary measures included: (1) time spent in open quadrants, (2) number of open quadrant entries, and (3) total transitions between zones. Increased open quadrant exploration was considered indicative of reduced anxiety‐like behavior.

#### 2.4.2 Open Field Test (PND 67–73)

The open field test was conducted to assess locomotor activity and exploratory behavior. Each animal was gently placed in one corner of a square arena (100 × 100 cm) under standardized illumination (60 lux). Behavior was recorded for 10 min using automated tracking software. We quantified: (1) total distance travelled, (2) time spent in the center zone (defined as > 15 cm from walls), and (3) number of center entries. Increased center time and entries were interpreted as indicators of reduced anxiety‐like behavior and enhanced exploration.

#### 2.4.3 Novel Object Recognition (PND 74–87)

The three‐phase NOR test assessed recognition memory over 24 h:

1. Familiarization (Trial 1): Animals explored two identical objects placed diagonally in the arena (8 min, 60 lux).
2. Novelty Preference (Trial 2): After a 2‐h inter‐trial interval, one familiar object was replaced with a novel object (8 min).
3. Memory Retention (Trial 3): Following 24 h, another novel object was introduced (8 min).

Exploration time (sniffing or touching within 2 cm) was recorded. The recognition index was calculated as follows:

$$\begin{array}{cc} & [\text{novel}\text{object}\text{exploration}\text{time}/(\text{novel}\\ & \;+\text{familiar}\text{object}\text{exploration}\text{time})]\times 100 \end{array}$$

Higher values indicate better recognition memory.

### 2.5 Cranial Magnetic Resonance Imaging (MRI, PND 53–69 and PND 137–151)

#### 2.5.1 Animal Preparation and Monitoring

MRI scans were conducted at two predefined developmental stages (early adolescence, PND 53–69 and adulthood, PND 137–151) to assess neurostructural changes associated with dietary interventions. Because of scheduling constraints and the number of animals, scan data were planned backwards from respective organ harvesting dates. Each animal underwent MRI scanning at both stages, in one of the following paired timepoint combinations: (1) PND 53–57 and PND 137–141; (2) PND 60–64 and PND 144–148; (3) PND 68–69 and PND 151. Animals were anesthetized using 5% isoflurane in O_2_/N_2_O (30:70) for induction, maintained at 1.5%–2.0% isoflurane, and positioned on a temperature‐regulated bed (37°C ± 0.5°C). Respiratory rate was continuously monitored.

#### 2.5.2 Imaging System

All scans were acquired using a 7T Biospec 70/20 MRI system (Bruker Biospin) equipped with:

1. A BGA12s high‐performance gradient system (450 mT/m maximum strength; Bruker Biospin).
2. An optimized cross‐coil configuration:
3. Transmit: 70‐mm inner diameter volume resonator (Bruker).
4. Receive: Anatomically shaped rat head surface coil (Rapid Biomedical).

#### 2.5.3 Image Acquisition

##### 2.5.3.1 Quantitative T_2_ Mapping

A multi‐slice multi‐echo (MSME) sequence was used with the following parameters: 1 mm slice thickness, 30 mm FOV, 128 × 128 matrix (230 µm in‐plane resolution), and 16 echo times (12.5–200 ms, 12.5 ms spacing). Total acquisition time was 7 min 40 sec. T_2_ relaxation maps were generated through pixel‐wise exponential fitting using MATLAB (R2020b, MathWorks).

##### 2.5.3.2 Diffusion Tensor Imaging (DTI)

A segmented spin‐echo EPI sequence (4 shots) was employed with:

1. 16 contiguous slices.
2. 30 diffusion directions (Jones30 scheme).
3. *b*‐value = 650 s/mm^2^ (plus 5 b_0_ images).
4. TE/TR = 28.1/3000 ms.

Total acquisition time was 7 min. Diffusion metrics were calculated using DTI‐Studio (Version 14.02.2017, Quelle: Citations | DSI Studio Documentation). Diffusion‐weighted images were processed to generate maps of fractional anisotropy (FA). FA measures the directionality of water diffusion, ranging from 0 (perfectly isotropic) to 1 (perfectly anisotropic), and serves as a marker for white matter microstructural integrity.

### 2.6 Blood Parameter Analysis

Blood samples were collected via retro‐orbital puncture under brief isoflurane anesthesia during (PND33‐35) and after the dietary intervention (PND146‐148) consistent with our established protocols. Following collection, EDTA plasma was promptly separated and transported to the Central Laboratory of the University Hospital Cologne for comprehensive analyses using standardized automated assays of clinical routine parameters (pH, pCO_2_, ctHb, sO_2_, Hctc, cK^+^, cNa^+^, cCa^+^, cCl^−^, cHCO3^−^, ABEc, cGlu, cLac, ALT, AST, total calcium, cholesterol, creatinine, hsCRP, HDL, PHOS, total protein, triglyceride, urea, cholinesterase, albumin).

### 2.7 Statistics

Statistical analyses of blood, metabolic, or morphological parameters as well as MRI‐data were performed using GraphPad Prism 9, with specific tests selected according to data distribution and experimental design. Additional statistical information for individual experiments is provided in the respective figure and table legends. For analyses of clinical basic parameters (i.e., body weights, 24‐h intake‐ and output monitoring, blood gas parameters, clinical chemistry parameters), non‐parametric Kruskal–Wallis tests followed by Dunn's post‐hoc comparisons were used, selected for their robustness to non‐normal distributions and small sample sizes. The data from the behavioral tests (NOR, elevated zero maze, and open field test) were analyzed in R 4.5 [28] with the package lmerTest 3.1 [29] using a linear mixed‐effects model including a random intercept for the run. For the NOR test, the analysis was structured to examine three predefined, hierarchically linked hypotheses: (1) Does the combined treatment with lutein, DHA, and choline improve NOR? (2) Is lutein necessary for this effect? (3) Is lutein alone sufficient to achieve the same effect? To obtain a more comprehensive behavioral profile, additional tests (elevated zero maze and open field test) were performed and analyzed using the same statistical approach as for the NOR data.

## 3 Results

### 3.1 Early Dietary Intervention With Lutein, DHA, and/or Choline Had no Significant Effect on Body Weight Progression or Parameters Measured by Intake‐ and Output Monitoring

None of the dietary interventions with lutein, DHA, and/or choline, either alone or in any combination, resulted in significant changes in body weight progression throughout the experiment or the parameters measured by intake‐ and output monitoring in adolescent [PND 40] or young adult (PND140) animals (Table 1). In detail, food intake, water intake, urinary excretion, and feces excretion remained unaffected across all experimental groups at both timepoints. The absence of any significant differences in these fundamental measures of energy balance and waste elimination indicates that the interventions did not alter the animals' basal metabolism. The stability in body weight, despite the supplementation, further supports that energy homeostasis was maintained. Consequently, the administered dietary factors did not impose a metabolically disruptive effect. In summary, the animals tolerated all dietary interventions well.

**TABLE 1:** Metabolic parameters at different time‐points.

		Control	Lutein	DHA	Choline	Lutein and DHA	Lutein and choline	DHA and choline	Lutein, DHA, and choline
		Body weight (g)							
PND2	m	7.24 ± 0.59	7.68 ± 0.59	7.37 ± 0.76	7.53 ± 0.76	7.36 ± 0.34	7.69 ± 0.44	7.34 ± 0.74	7.25 ± 0.36
	f	6.99 ± 0.31	6.84 ± 0.70	7.13 ± 0.52	7.05 ± 0.56	6.88 ± 0.56	6.96 ± 0.62	6.93 ± 0.73	7.04 ± 0.54
PND14	m	36.19 ± 1.19	37.35 ± 1.78	36.74 ± 3.29	35.09 ± 2.66	36.46 ± 2.55	37.10 ± 2.28	39.34 ± 2.66	36.59 ± 2.21
	f	34.15 ± 4.10	35.56 ± 1.02	36.94 ± 1.58	34.86 ± 2.28	35.10 ± 2.79	35.38 ± 3.10	38.24 ± 1.66	34.50 ± 3.31
PND21	m	66.14 ± 3.99	70.60 ± 3.53	68.75 ± 7.16	65.11 ± 5.64	68.39 ± 7.31	67.31 ± 5.91	70.53 ± 5.29	67.15 ± 3.12
	f	63.85 ± 4.41	64.19 ± 2.63	66.36 ± 4.38	61.66 ± 5.16	62.77 ± 6.26	62.26 ± 7.65	67.35 ± 4.56	61.84 ± 5.02
PND56	m	339.43 ± 30.30	343.13 ± 26.06	350.43 ± 27.58	324.00 ± 41.60	336.29 ± 22.57	348.43 ± 17.82	351.29 ± 19.70	340.38 ± 22.15
	f	238.25 ± 10.11	229.83 ± 17.03	239.50 ± 21.83	224.00 ± 11.68	227.13 ± 18.39	226.13 ± 15.25	236.25 ± 22.31	226.00 ± 25.03
PND105	m	498.25 ± 50.21	528.38 ± 38.90	519.75 ± 46.00	504.63 ± 47.44	491.50 ± 46.45	543.00 ± 27.19	520.14 ± 30.38	510.88 ± 40.91
	f	305.57 ± 14.32	295.86 ± 24.48	308.25 ± 21.59	288.13 ± 7.75	294.67 ± 19.33	296.50 ± 17.68	308.75 ± 32.03	292.38 ± 28.31
Sacrifice	m	556.88 ± 63.63	596.89 ± 44.54	581.00 ± 54.65	569.50 ± 54.70	544.83 ± 60.40	607.29 ± 32.68	576.57 ± 39.21	579.14 ± 24.88
	f	318.17 ± 21.16	320.14 ± 32.83	329.88 ± 22.19	308.88 ± 10.04	323.00 ± 23.18	324.38 ± 18.48	335.50 ± 38.55	310.13 ± 21.62

		Metabolic parameters PND40							
Food intake	m	34.6 ± 4.62	34.6 ± 4.16	35.5 ± 3.26	35.9 ± 3.82	34.3 ± 4.85	34.1 ± 3.52	35.0 ± 3.56	34.6 ± 3.93
	f	30.4 ± 3.60	29.0 ± 5.62	30.8 ± 6.84	28.6 ± 4.06	24.5 ± 6.03	26.2 ± 4.06	28.1 ± 7.33	27.0 ± 5.02
Water intake	m	18.7 ± 4.36	19.6 ± 3.65	18.0 ± 3.05	18.7 ± 2.92	17.1 ± 3.30	17.5 ± 7.32	20.8 ± 3.80	17.6 ± 4.22
	f	19.2 ± 6.09	19.0 ± 3.40	22.7 ± 9.68	15.7 ± 2.69	17.4 ± 3.56	15.5 ± 3.55	17.1 ± 5.62	17.5 ± 3.94
Urinary excretion	m	8.53 ± 3.51	10.7 ± 2.35	10.1 ± 2.29	8.58 ± 2.49	9.35 ± 2.95	8.51 ± 3.60	11.0 ± 3.21	8.31 ± 2.59
	f	10.5 ± 4.70	10.1 ± 2.37	12.7 ± 7.41	6.59 ± 2.72	8.80 ± 3.98	8.13 ± 2.60	8.32 ± 1.63	8.25 ± 2.63
Feces excretion	m	3.18 ± 0.52	3.51 ± 1.22	3.21 ± 0.67	3.96 ± 1.35	3.72 ± 0.90	3.52 ± 0.91	3.38 ± 1.04	3.36 ± 0.68
	f	2.91 ± 0.65	2.79 ± 0.45	2.67 ± 0.65	2.70 ± 0.72	2.49 ± 1.02	2.41 ± 0.91	3.58 ± 1.30	2.73 ± 1.11

		Metabolic parameters PND140							
Food intake	m	23.7 ± 7.77	24.7 ± 4.40	25.8 ± 3.58	25.2 ± 2.54	21.2 ± 8.25	19.2 ± 5.56	21.8 ± 5.10	21.9 ± 6.37
	f	22.3 ± 2.83	18.6 ± 3.63	20.7 ± 5.57	19.9 ± 5.56	18.0 ± 4.78	18.8 ± 4.35	20.2 ± 3.04	21.6 ± 3.94
Water intake	m	24.7 ± 7.73	26.3 ± 5.83	26.6 ± 3.32	23.4 ± 3.35	18.1 ± 11.0	22.0 ± 7.73	27.6 ± 11.8	21.3 ± 6.51
	f	25.1 ± 4.36	21.5 ± 6.52	24.7 ± 5.21	21.8 ± 3.67	18.6 ± 5.43	20.8 ± 6.06	21.5 ± 4.79	27.5 ± 12.0
Urinary excretion	m	11.3 ± 3.27	12.1 ± 3.74	11.7 ± 1.53	11.3 ± 2.60	9.36 ± 2.17	12.1 ± 5.07	10.6 ± 2.42	11.2 ± 3.56
	f	9.56 ± 2.12	10.6 ± 2.55	11.0 ± 3.22	9.98 ± 3.53	7.86 ± 4.05	9.51 ± 3.70	12.1 ± 6.91	16.4 ± 6.65
Feces excretion	m	2.45 ± 1.06	2.94 ± 1.14	2.30 ± 0.74	3.33 ± 1.46	2.63 ± 1.44	2.41 ± 1.28	2.67 ± 1.06	2.66 ± 0.74
	f	2.21 ± 1.08	2.63 ± 0.66	1.94 ± 1.42	1.63 ± 0.94	2.81 ± 1.26	2.34 ± 0.48	2.85 ± 0.97	2.79 ± 0.92

*Note*: This table presents the body weight measurements at key postnatal days [PND2, PND14, PND21, PND56, PND105, and sacrifice (PND152‐160)] and metabolic parameters (food intake, water intake, fecal, and urinary excretion) measured for 24 h at PND36‐43 and PND137‐142 for male (m) and female (f) rats across eight dietary intervention groups: Control, Lutein, DHA, Choline, and their respective combinations (*n* = 6–8 per group). Values represent mean ± SD. Statistical analysis using a mixed‐effect model and post hoc Tukey test for multiple comparisons against the control revealed no significant differences.

### 3.2 Early Dietary Intervention With Lutein, DHA, and Choline Did Not Alter Anxiety‐Like Behavior in Female Rats

**FIGURE 2:**
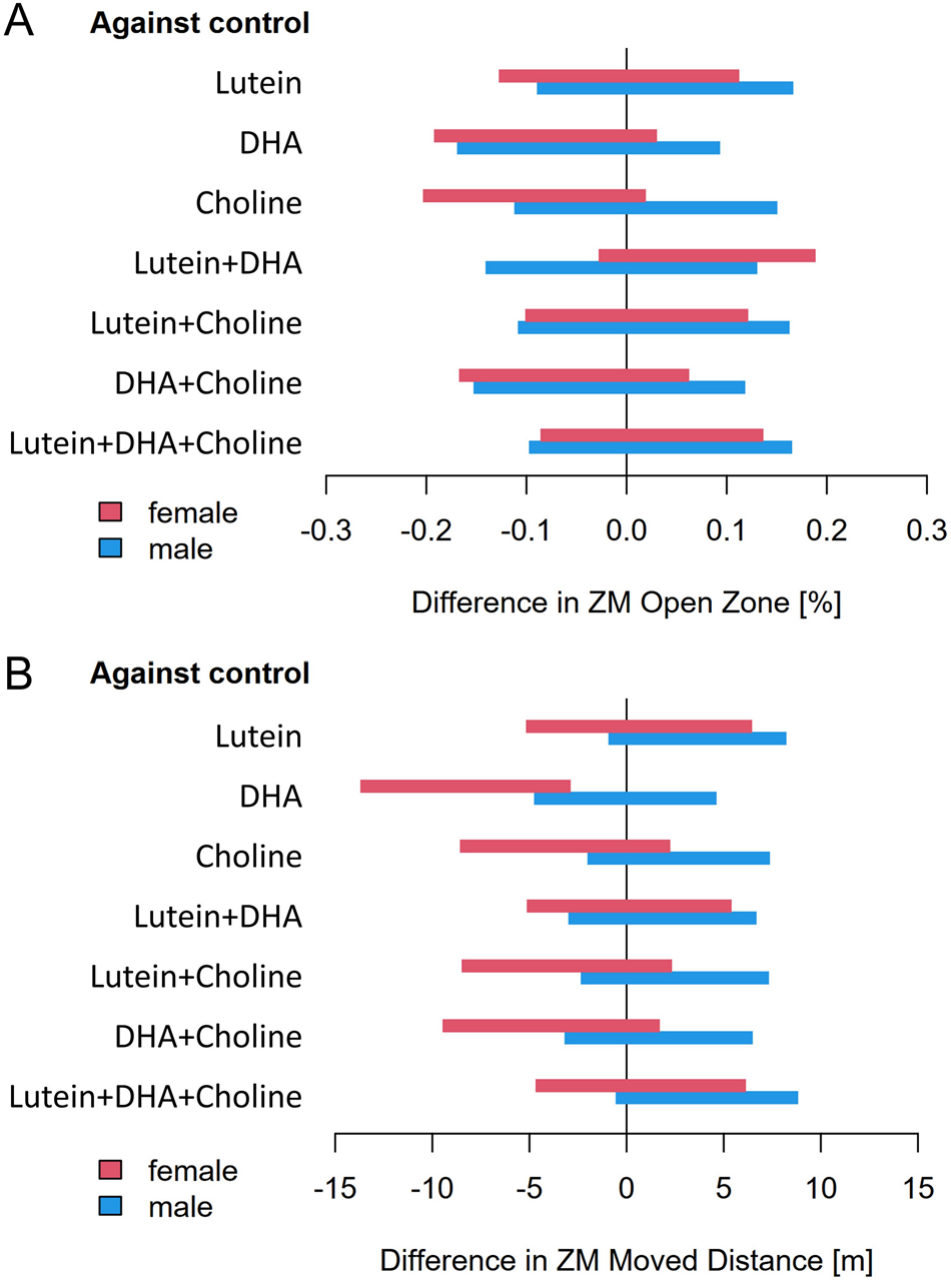
Impact of lutein, DHA, and choline on anxiety‐like behavior, as assessed in the elevated zero maze. (A) Difference in percentage (%) of time spent in the open zone relative to control. (B) Difference in moved distance (m) relative to control. Data represent linear mixed‐effects model estimates (with 95% confidence intervals) for female (red) and male (blue) rats receiving single nutrients or every possible combination.

Linear mixed‐effects model analysis of the elevated zero maze data (collected 8–10 days after the end of the dietary intervention) showed no significant effects of the dietary treatment on anxiety‐like behavior when tested against our predefined hypotheses (Figure 2). Specifically, neither the combined lutein+DHA+choline regimen, the necessity of lutein within this combination, nor lutein supplementation alone altered the time spent in the open zone or the total distance moved.

### 3.3 Early Dietary Intervention With Lutein, DHA, and Choline Had Limited Impact on Locomotor Activity and no Relevant Impact on Exploratory Behavior in Offspring Rats

**FIGURE 3:**
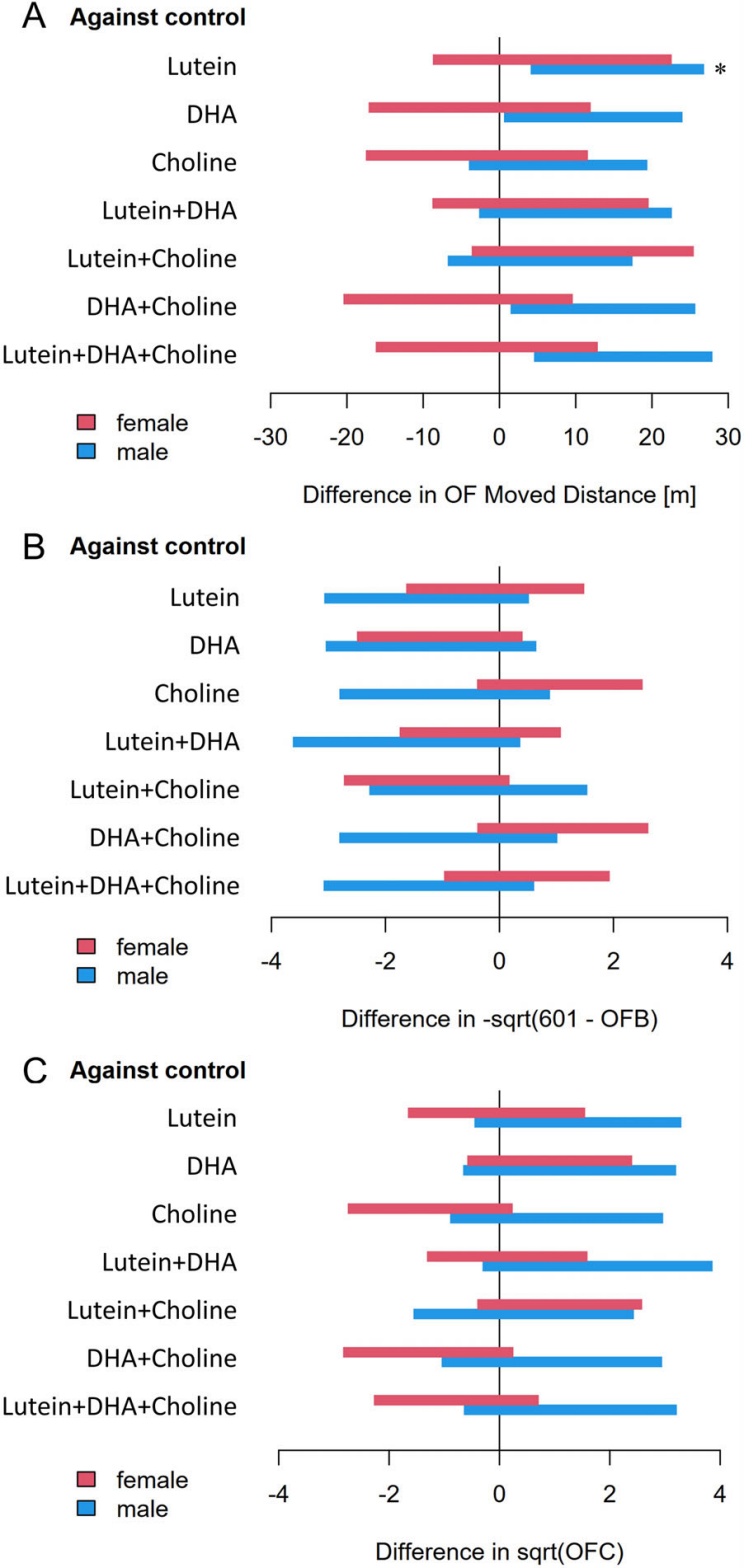
Impact of lutein, DHA, and choline on locomotor activity and exploratory behavior, as assessed in the open field test. Linear mixed‐effects model estimates (95% confidence intervals) of treatment effects relative to control for female (red) and male (blue) rats receiving single nutrients or every possible combination. (A) Difference in total moved distance (m). (B) Difference in time spent in the border area, expressed as negative square root (601—border time). (C) Difference in time spent in the center, expressed as square root of center time. Statistical significance is depicted by asterisks (*, *p* < 0.05).

Linear mixed‐effects model analysis of data from the open field test (collected 11–17 days after the end of the dietary intervention) showed no significant effects of dietary treatment on anxiety‐like behavior when tested against our predefined hypotheses. Specifically, neither the combined treatment with lutein, DHA, and choline, nor the necessity of lutein within this combination, nor lutein supplementation alone altered time spent in the open or border zones (Figure 3B,C). The only statistically significant finding was that male rats receiving lutein alone exhibited increased locomotor activity compared to controls (95% CI: 4.09 to 26.83, *p* < 0.05; Figure 3A). This isolated effect of lutein monotherapy did not translate into altered exploratory behavior.

### 3.4 Combined Early Dietary Intervention With Lutein, DHA, and Choline Enhances Recognition Memory in Female Rats

**FIGURE 4:**
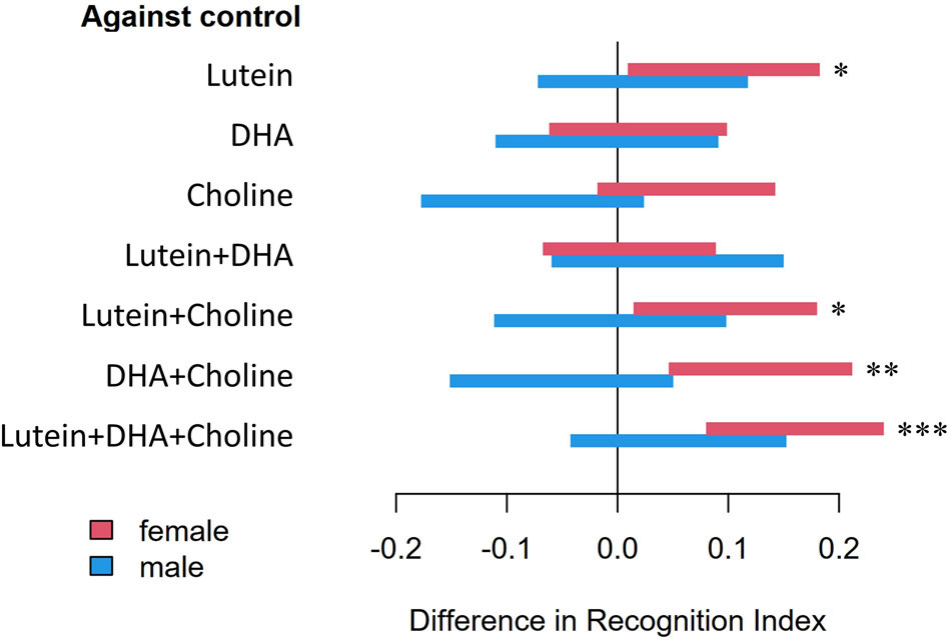
Sex‐specific enhancement of recognition memory by perinatal lutein, DHA, and choline supplementation. Linear mixed‐effects model estimates (with 95% confidence intervals) of the treatment effect on the recognition index relative to control for female (red) and male (blue) rats receiving single nutrients or all possible combinations. The recognition index was calculated as: [novel object exploration time/(novel + familiar object exploration time)] × 100. Statistical significance is depicted by asterisks (*, *p* < 0.05; **, *p* < 0.01; ***, *p* < 0.001).

Analysis of NOR performance (assessed 18–31 days after the end of the dietary intervention) revealed a distinct, sex‐specific pattern of cognitive modulation in early adulthood. Linear mixed‐effects modeling showed that all significant treatment effects on recognition memory were confined to female rats, since no intervention significantly altered NOR performance in male rats (all *p* > 0.05; Figure 4).

In females, the greatest improvement based on the effect size was observed following the combined administration of lutein, DHA, and choline. Compared to the control group, this triple intervention significantly improved novel object discrimination (95% CI: 0.08 to 0.24, *p* < 0.001; Figure 4), strongly supporting our primary hypothesis of enhanced cognitive benefits from combined nutrient supplementation. Further analysis of our predefined hypotheses revealed that lutein is not a necessary component for this improvement. The combination of DHA and choline also produced a significant, however weaker, enhancement in recognition memory compared to controls (95% CI: 0.046 to 0.212; *p* < 0.01; Figure 4). Lutein administered as a monotherapy was itself sufficient to improve performance in females relative to the control diet (95% CI: 0.009 to 0.183, *p* < 0.05; Figure 4). However, direct statistical comparisons aimed at isolating a unique yet superior effect of lutein beyond that of other treatments did not reach significance.

Taken together, these findings demonstrate that early dietary supplementation can selectively influence cognitive phenotypes in a sex‐dependent manner. Exposure to lutein, DHA, and choline—individually or in combination—improves recognition memory exclusively in female offspring. The greatest efficacy was observed with the triple combination, suggesting a possible combined effect and highlighting the complex interplay of these bioactive food components during neurological development, particularly during the weaning period.

### 3.5 Early Dietary Intervention With Lutein, DHA, and/or Choline Had Only Minor Impact on Routine Blood Parameters

While our study revealed significant behavioral modifications, the corresponding changes in blood gas, electrolyte, inflammatory, and metabolic parameters were remarkably limited. At PND35, male rats with early dietary interventions exhibited the following significant but physiologically irrelevant alterations compared to male controls: choline supplementation alone slightly increased aspartate aminotransferase (AST) plasma concentrations, while combined choline and DHA supplementation slightly elevated high‐density lipoprotein (HDL). All treatment combinations containing choline slightly increased total protein levels. Further, some groups of male and female rats with early dietary interventions demonstrated a few significant, but again physiologically irrelevant changes in some electrolytes (see Tables S1 and S2 for complete data). On day 140 after birth (PND140), some changes in these metabolic patterns occurred. Male rats that had been given a combination of choline and lutein now showed slightly reduced, but physiologically irrelevant, plasma concentrations of alanine aminotransferase (ALT). Since the total calcium concentration in the male control animals was at the upper limit of the normal range, the total calcium concentrations in several intervention groups were significantly lower (DHA, DHA + choline, lutein + DHA, lutein + choline, lutein + DHA + choline), while total protein was also specifically lower in the lutein + choline group. These findings (full data in Tables S3 and S4) suggest some developmental transitions in nutrient metabolism, but most of these have no physiological relevance.

### 3.6 Early Dietary Intervention With Lutein, DHA, and/or Choline Had No Significant Effect on Brain Growth and Morphology

Despite the varying results of the behavioral tests, we observed that the morphological parameters of central nervous system development were not influenced by early nutritional intervention at any point in time or in any of the interventions. Brain weight and volume showed no statistically significant differences between groups (*p* > 0.05 for all comparisons). The same applied to the weight of the other organs (Table 2) and body weight (Table 1), which overall indicates that there were no systemic growth changes. Longitudinal MRI analyses at PND53‐69 and PND137‐151 revealed no intervention effects on structural brain development: whole‐brain volumes were comparable across all groups (*p* > 0.05), with no differences in growth trajectories between PND53‐69 and PND137‐151. DTI detected no microstructural changes in the hippocampus, cingulate cortex, or corpus callosum (Table 2). More specifically, no differences in FA were detected between the intervention groups, measuring the directionality of water diffusion and thereby serving as a marker for white matter integrity. These results suggest that although specific behavioral changes were observed, the intervention did not cause any changes in gross somatic or neuroanatomical development neither influencing anisotropy.

**TABLE 2:** Organ weights, brain volume, and fractional anisotropy values for brain compartments.

		Control	Lutein	DHA	Choline	Lutein and DHA	Lutein and choline	DHA and choline	Lutein, DHA, and choline
		Organ weights (g)							
Brain	m	2.22 ± 0.16	2.20 ± 0.14	2.20 ± 0.09	2.14 ± 0.07	2.22 ± 0.14	2.17 ± 0.09	2.22 ± 0.16	2.20 ± 0.12
	f	2.05 ± 0.09	2.07 ± 0.11	2.01 ± 0.05	2.03 ± 0.09	2.03 ± 0.05	2.04 ± 0.11	2.04 ± 0.09	2.05 ± 0.09
Liver	m	16.59 ± 2.46	18.00 ± 2.77	17.45 ± 2.98	16.76 ± 2.19	16.60 ± 1.82	18.69 ± 2.58	17.06 ± 2.85	19.07 ± 1.97
	f	8.82 ± 0.49	9.19 ± 1.39	9.42 ± 0.75	9.23 ± 0.62	9.36 ± 1.22	9.26 ± 0.88	9.69 ± 1.45	9.04 ± 1.57
Retroperitoneal Fat	m	12.82 ± 4.38	19.56 ± 7.30	14.55 ± 3.14	14.45 ± 6.06	13.21 ± 3.41	15.91 ± 3.90	14.20 ± 3.54	16.84 ± 4.44
	f	4.31 ± 0.98	4.87 ± 1.93	5.91 ± 3.03	4.53 ± 1.32	4.81 ± 1.41	5.38 ± 1.85	5.46 ± 2.01	3.67 ± 1.25
Right kidney	m	1.59 ± 0.25	1.66 ± 0.19	1.73 ± 0.26	1.65 ± 0.21	1.62 ± 0.19	1.70 ± 0.15	1.69 ± 0.13	1.59 ± 0.15
	f	1.43 ± 0.15	1.36 ± 0.21	1.58 ± 0.22	1.44 ± 0.16	1.30 ± 0.21	1.45 ± 0.25	1.43 ± 0.23	1.35 ± 0.21
Left kidney	m	1.55 ± 0.25	1.58 ± 0.20	1.64 ± 0.30	1.60 ± 0.23	1.58 ± 0.20	1.69 ± 0.11	1.65 ±0.15	1.52 ± 0.14
	f	1.37 ± 0.09	1.32 ± 0.19	1.48 ± 0.23	1.34 ± 0.11	1.30 ± 0.19	1.47 ± 0.37	1.32 ± 0.32	1.34 ± 0.27
Adrenal gland	m	0.50 ± 0.01	0.05 ± 0.01	0.05 ± 0.02	0.05 ± 0.01	0.05 ± 0.02	0.08 ± 0.02	0.05 ± 0.02	0.07 ± 0.03
	f	0.08 ± 0.03	0.07 ± 0.02	0.07 ± 0.03	0.07 ± 0.02	0.06 ± 0.02	0.07 ± 0.01	0.05 ± 0.01	0.06 ± 0.02
Mesenteric fat	m	6.78 ± 2.29	7.67 ± 2.79	7.51 ± 1.19	7.31 ± 2.80	6.03 ± 1.15	7.37 ± 1.32	6.44 ± 2.80	7.95 ± 0.93
	f	4.27 ± 0.80	3.94 ± 0.78	4.32 ± 1.10	3.59 ± 0.79	4.15 ± 1.04	4.53 ± 4.53	4.02 ± 1.27	3.33 ± 1.07
Epididymal fat	m	13.13 ± 4.22	15.39 ± 3.68	14.18 ± 2.13	13.14 ± 3.73	12.86 ± 2.72	16.24 ± 2.25	13.10 ± 3.49	15.61 ± 2.35
Testicle	m	1.68 ± 0.36	1.81 ± 0.32	2.00 ± 0.41	1.96 ± 0.31	1.77 ± 0.37	1.88 ± 0.22	1.85 ± 0.39	1.94 ± 0.22

		Brain volume (mm^3^)							
Brain volume PND60	m	2406 ± 79	2395 ± 123	2374 ± 87	2343 ± 75	2424 ± 116	2350 ± 49	2404 ± 118	2378 ± 73
	f	2281 ± 89	2268 ± 99	2235 ± 66	2213 ± 108	2239 ± 77	2204 ± 58	2216 ± 99	2242 ± 78
Brain volume PND150	m	2610 ± 143	2567 ± 176	2601 ± 150	2487 ± 162	2647 ± 125	2577 ± 80	2654 ± 149	2561 ± 81
	f	2448 ± 84	2449 ± 107	2402 ± 66	2400 ± 79	2443 ± 61	2380 ± 79	2422 ± 110	2451 ± 97

		Fractional anisotropy [0 (perfectly isotropic) to 1 (perfectly anisotropic)] PND 150							
FA hippocampus	m	0.15 ± 0.02	0.16 ± 0.02	0.18 ± 0.02	0.17 ± 0.03	0.15 ± 0.02	0.18 ± 0.05	0.17 ± 0.01	0.17 ± 0.02
	f	0.17 ± 0.02	0.17 ± 0.04	0.19 ± 0.03	0.17 ± 0.01	0.17 ± 0.04	0.19 ± 0.02	0.17 ± 0.03	0.17 ± 0.02
FA cingulate cortex	m	0.18 ± 0.03	0.18 ± 0.02	0.19 ± 0.05	0.17 ± 0.02	0.19 ± 0.01	0.17 ± 0.02	0.17 ± 0.02	0.16 ± 0.14
	f	0.16 ± 0.01	0.16 ± 0.01	0.15 ± 0.02	0.16 ± 0.02	0.16 ± 0.02	0.15 ± 0.004	0.16 ± 0.02	0.17 ± 0.02
FA corpus callosum	m	0.53 ± 0.06	0.53 ± 0.05	0.58 ± 0.07	0.58 ± 0.03	0.57 ± 0.04	0.55 ± 0.04	0.59 ± 0.03	0.56 ± 0.02
	f	0.55 ± 0.05	0.55 ± 0.02	0.58 ± 0.03	0.52 ± 0.03	0.55 ± 0.04	0.56 ± 0.04	0.54 ± 0.05	0.57 ± 0.03

*Note*: This table presents organ weights, brain volume (PND60 and PND150), and fractional anisotropy values (PND150) for male (m) and female (f) rats (except epididymal fat and testicles only for males) across eight dietary intervention groups: Control, Lutein, DHA, Choline, and their respective combinations (*n* = 4–9 per group). Values represent mean ± SD. Statistical analysis using a mixed‐effect model and post‐hoc Tukey test for multiple comparisons against the control revealed no significant differences.

## 4 Discussion

Our study systematically examined the individual and combined effects of a developmental dietary intervention with lutein, DHA, and choline on neurobehavioral and neuroanatomical outcomes. Using a comprehensive nutritional approach that included the use of all dietary factors individually and in all possible combinations, we evaluated the post‐intervention effects on recognition memory, anxiety‐like behaviors, locomotor activity, and brain maturation using MRI in a rodent model. Our approach allowed us to simultaneously investigate both nutrient‐specific and potential combined effects, and provided new insights into how these bioactive compounds influence neurodevelopment when administered during the critical growth periods surrounding weaning.

Our study revealed two striking patterns of neurobehavioral imprinting. First, the recognition memory‐enhancing effects of our perinatal nutritional intervention were strictly sex‐specific, with relevant significant improvements exclusively in female offspring. Second, while lutein monotherapy was able to modulate cognitive performance, the most robust behavioral phenotype resulted from the additive effect of lutein, DHA, and choline, underscoring the superior efficacy of this combinatorial approach during neurodevelopment.

The pronounced influence of lutein on behavioral phenotype and recognition memory in our study is consistent with its unique properties for neurodevelopment. As one of the most abundant carotenoids in the human diet [30] and a potent antioxidant [31], lutein is able to cross the blood–brain barrier and accumulate in regions associated with behavior like the cerebellum, the frontal cortex, or the occipital cortex [32]. Interestingly, it accounts for approximately 60% of the carotenoids in the brains of infants, even though it only accounts for 12% of the carotenoids in their diet. This highlights its selective neuronal uptake and potential functional importance during critical developmental stages, which is particularly significant given that premature infants are deficient in lutein [33, 34]. In vitro studies highlight positive effects of lutein on membrane stability [35], while other studies also demonstrate upregulation of antioxidant enzymes and secretion of anti‐inflammatory cytokines with simultaneous downregulation of reactive oxygen species (ROS) production [24]. In a mouse model for multiple sclerosis, a significant improvement in remyelination was demonstrated through a reduction in neuroinflammation and simultaneous upregulation of myelin‐forming genes [36]. Furthermore, an animal study with a design comparable to ours also reported lutein‐induced improvements in passive avoidance, object recognition, and spatial memory, with the antioxidant properties of lutein identified as a potential mechanism for lutein's efficacy [37]. An observational study in humans concluded that higher lutein intake during childhood correlates with better performance on the Peabody Picture Vocabulary Test (PPVT‐II), suggesting improved language development [14]. Moreover, a randomized double‐blind placebo controlled clinical trial in adults found that lutein and zeaxanthin supplementation significantly improved visual episodic memory and learning [38]. The consistency of our findings with these cross‐species observations supports the plausibility of lutein's role as an important micronutrient for neurological development and highlights its potential as a nutritional strategy to support cognitive health during critical developmental stages.

Lutein's potent antioxidant properties likely contribute to its neuroprotective effects during early development, a period characterized by both exceptional plasticity and heightened metabolic vulnerability [39]. Although the developing brain displays remarkable resilience to stressors [40], it undergoes rapid growth, which is mainly driven by oxidative metabolism. This leads to oxygen consumption that is almost twice as high as in older children and thus to increased production of free radicals [40]. At the same time, newborns have relatively low levels of endogenous antioxidant enzymes. Therefore, the antioxidant properties of lutein can provide crucial protection during this sensitive phase [4, 41, 42, 43]. This protective mechanism highlights the potential of early childhood nutritional strategies to optimize neurological development.

As already mentioned, not only did lutein have an effect on recognition memory, but DHA and choline also improved the recognition of new objects, both in combination with each other and in combination with lutein, or in a triple combination. Enhancing effects of dietary components have been described previously, with DHA and choline in particular exhibiting well‐documented additive properties when administered together. A study in rodents showed a higher number of hippocampal neurons when DHA and choline were administered together compared to individual supplementation [44]. In a clinical study, a similar direction of interaction between lutein, DHA, and choline was reported by Cheatham et al. [13], where both the combination of lutein and choline or DHA and choline resulted in a beneficial effect on recognition memory (measured using an EEG response to new vs. familiar stimuli). Thus, this clinical evidence provides support for potential value of the current study.

Beyond the specific effects of lutein, our results show a pronounced sex dimorphism in the response to lutein supplementation during development, with relevant cognitive and behavioral improvements observed exclusively in female offspring. This sex specificity suggests fundamental differences in lutein metabolism, antioxidant pathway activation, or neurodevelopmental sensitivity between males and females. The marked improvements in recognition memory in female animals suggest that lutein may preferentially modulate the neural circuits that control emotion regulation and cognitive functions in a sex‐specific manner.

These findings are consistent with compelling evidence demonstrating significant differences in antioxidant metabolic pathways between the sexes, with females having a significantly more efficient antioxidant system and better defenses than males [45, 46, 47]. In particular, an observational study in humans found that female newborns have higher total antioxidant levels and lower plasma hydroperoxides compared to male newborns, along with higher antioxidant enzyme activity [48]. The antioxidant advantage appears to begin in the womb, as mothers who carry female fetuses also have higher antioxidant status and reduced inflammatory markers such as interleukin‐6 (IL‐6), tumor necrosis factor alpha (TNF‐α), and prostaglandin E2 [48]. Further studies confirm increased oxidative stress or increased production of ROS in males compared to females, as observed in flies [49], rats [50], or healthy adult humans [51]. Given these proven sex differences in dealing with oxidative stress, the antioxidant properties of lutein could be particularly beneficial for women by supplementing their already enhanced—but potentially more stressed—antioxidant system. This mechanism could explain why females in our study responded better to lutein supplementation, especially during the high metabolic demands of neural circuit development and synaptic plasticity.

Regarding DHA, several studies have reported a stronger beneficial effect of DHA supplementation in females compared to males [52, 53]. While the underlying mechanisms remain elusive, this sex‐specific effect may be partially explained by differences in the incorporation of DHA into phospholipid membranes. When equal doses of DHA are administered to both sexes, the same absolute amount of DHA may result in a relatively higher incorporation into cell membranes in females [53]. This could lead to more pronounced functional effects despite identical supplementation regimens. Interestingly, similar sex‐specific considerations may apply to choline. Lower choline blood levels have been correlated with obesity and higher BMI in humans [54, 55], suggesting that body composition (which is different in males and females) could also influence choline bioavailability and its physiological effects. Furthermore, if females exhibit greater membrane incorporation of DHA, the combined transport mechanism for DHA and choline may enhance choline uptake when both nutrients are administered together. However, further studies are needed to directly test these hypotheses and to investigate other potential mechanisms underlying sex differences, including differential metabolism of DHA and choline, transport across the blood–brain‐barrier, or estrogen‐mediated effects. Regarding the latter, estrogen has been shown to upregulate enzymes involved in DHA incorporation and choline uptake into membranes, such as elongases and desaturases [52, 56, 57].

Our findings have significant translational potential for nutritional strategies aimed at optimizing neurological development, particularly during critical developmental periods. The pronounced sex‐specific response underscores the need to consider sex as a critical variable in both nutritional research and clinical recommendations. Girls may derive greater benefit from antioxidant‐rich interventions at a young age. The dominant role of lutein in mediating neurobehavioral outcomes underscores its potential as an important micronutrient for supplementation programs during infancy. Its preferential accumulation in brain and nerve tissue, combined with its multimodal mechanisms including antioxidant protection, anti‐inflammatory effects, and membrane stabilization, make lutein a particularly promising candidate for nutritional prevention or alleviation of neurological developmental disorders. From a clinical perspective, this could be particularly relevant for premature babies, who lack the lutein reservoir built up in the third trimester and are exposed to increased oxidative stress [33]. The demonstrated additive effects of DHA and choline in supporting hippocampal development, combined with the antioxidant and anti‐inflammatory properties of lutein, points to the potential superiority of multi‐nutrient approaches for comprehensive support of neurological development. This is further supported by the fact that all three substances are components of human breast milk [13]. This combination strategy may be particularly valuable in addressing the complex pathophysiology of neurological developmental disorders.

Nevertheless, our study has some limitations that we would like to point out. One limitation is that we did not measure blood or tissue concentrations of lutein, DHA, or choline. Retrospective quantification was not feasible due to the time that has elapsed since the project's completion and the marked instability of lutein and DHA (particularly their susceptibility to oxidation) during prolonged storage. It is important to note that our primary goal was to ensure consistently adequate dietary availability to establish a causal link between lutein and/or DHA supplementation during a critical phase of early brain development and subsequent cognitive performance. The controlled dietary intervention supports the validity of the effects we observed. Of course, it would be highly interesting to elucidate the underlying molecular mechanisms in future studies. Another limitation of our study is the timing of the behavioral assessments (PND 74–87), which took place 18–31 days after the end of the dietary intervention. While this corresponds to (early) adulthood in rats, the long‐term persistence of the observed effects beyond this point still needs to be demonstrated in future studies. We also note that our study was not designed as a comprehensive mechanistic investigation. Although we performed some exploratory histological analyses to gain a rough picture of potential cellular correlates [58], we did not measure antioxidant enzyme activity, lipid peroxidation markers, or sex hormone profiles. Consequently, our interpretation of the observed female‐specific effects, particularly the plausible role of estrogen‐mediated neuroprotection, remains speculative. However, due to complete consumption of hippocampal tissue samples and the instability of these analytes after a longer period of time, retrospective measurements are not feasible. Future studies should include direct measurements of these parameters to fully elucidate the underlying mechanisms.

In conclusion, we note that despite behavioral improvements, no significant macro‐ or microstructural changes were detected using MRI or DTI. However, the absence of imaging changes does not invalidate the behavioral findings. Behavioral changes may be attributable to subtle adaptations at the synaptic, molecular, or neurotransmitter level that lie below the resolution limit of conventional structural imaging [59, 60]. DTI is primarily sensitive to major white matter alterations and may lack the resolution to detect subtle, region‐specific changes. Moreover, functional rather than structural neural adaptations are not captured by structural MRI or DTI. Future studies should employ complementary functional and molecular approaches to further elucidate the neural substrates of the observed behavioral effects.

In summary, our combinatorial nutrient approach demonstrates that recognition memory can be enhanced by supplementation with multiple nutrients, highlighting the complex interactions between bioactive compounds in the diet. We demonstrate that lutein acts as an important driver of improvements in cognitive function, as seen by enhancement of recognition memory. Our results advocate for the development of sex‐specific nutritional recommendations during critical developmental periods and support the inclusion of lutein in maternal, infant, and childhood supplementation regimens. Future clinical trials should investigate whether lutein supplementation, particularly in combination with DHA and choline, can improve neurocognitive outcomes in at‐risk populations such as preterm infants or children with neurodevelopmental disorders, while accounting for potential sex‐specific responses.

## Author Contributions

**Leon Saschin**: data curation, formal analysis, investigation, validation, visualization, writing – original draft, and writing – review and editing. **Gregor Fink**: conceptualization, data curation, formal analysis, investigation, methodology, visualization, and writing – original draft. **Maria Wohlfarth**: investigation and methodology. **Jonas Hauser**: conceptualization, data curation, formal analysis, and resources. **Jian Yan**: conceptualization, data curation, formal analysis, and resources. **Dirk Wiedermann**: formal analysis, methodology, and visualization. **Mathias Hoehn**: formal analysis, methodology, and visualization. **Jochen Wilhelm**: formal analysis and visualization. **Lea Quell**: writing – original draft and writing – review and editing. **Elisabeth Sous**: data curation and formal analysis. **Jenny Voggel**: writing – original draft. **Jörg Dötsch**: project administration and supervision. **Eva Nüsken**: conceptualization, data curation, funding acquisition, investigation, project administration, supervision, and writing – review and editing. **Kai‐Dietrich Nüsken**: conceptualization, data curation, funding acquisition, investigation, methodology, project administration, supervision, and writing – review and editing. All authors read and approved the final article.

## Funding

The authors declare that financial support was received for the research. This study was supported by a grant from Nestlé Société des Produits Nestlé, SA, Switzerland.

## Conflicts of Interest

J.H. is an employee of Société des Produits Nestlé, S.A., Switzerland. J.Y. was during the time the study was conducted an employee of Société des Produits Nestlé, S.A., Switzerland. This study was in part supported by Nestlé Research, Société des Produits Nestlé, SA, Switzerland. The remaining authors declare that the research was conducted without any commercial or financial relationship that could be construed as a potential conflict of interest.

## Supporting information




**Supporting File**: mnfr70589‐sup‐0001‐SuppMat.docx.

## Data Availability

All data supporting the findings of this study are available within the paper and its Supporting Information files. Should any raw data files be needed in another format they are available from the corresponding author upon request.
