# Supplementary material for: Dietary Intervention With Lutein, Docosahexaenoic Acid, and Choline During Early Development Improves Recognition Memory in Young Adult Females in an Experimental Model
Source: Mol Nutr Food Res. 2026 Aug 27;70(17):e70589. doi: 10.1002/mnfr.70589 (PMC13522655; doi:10.1002/mnfr.70589)
Supplement: Supplementary file 1 [file MNFR-70-e70589-s001.docx]

**Supplementary Table 1:** **Blood gas analysis at postnatal day 33-35.**

|  | | **Control** | **Lutein** | **DHA** | **Choline** | **Lutein**  **DHA** | **Lutein**  **Choline** | **DHA**  **Choline** | **Lutein**  **DHA**  **Choline** |
| --- | --- | --- | --- | --- | --- | --- | --- | --- | --- |
| **pH** | m | 7.45 ± 0.04 | 7.45 ± 0.07 | 7.48 ± 0.11 | 7.40 ± 0.06 | 7.44 ± 0.05 | 7.46 ± 0.07 | 7.45 ± 0.06 | 7.43 ± 0.02 |
|  | f | 7.40 ± 0.04 | 7.45 ± 0.07 | 7.45 ± 0.07 | 7.42 ± 0.05 | 7.45 ± 0.03 | 7.44 ± 0.02 | 7.44 ± 0.06 | 7.45 ± 0.04 |
| **pCO_2_**  **[mmHg]** | m | 38.1 ± 6.20 | 38.6 ± 9.23 | 37.7 ± 9.64 | 44.1 ± 10.2 | 40.5 ± 6.71 | 36.2 ± 8.55 | 37.0 ± 7.85 | 41.1 ± 4.39 |
|  | f | 43.8 ± 7.15 | 37.6 ± 8.63 | 39.0 ± 10.50 | 40.9 ± 7.19 | 37.3 ± 4.19 | 35.9 ± 6.78 | 38.1 ± 6.08 | 38.6 ± 7.25 |
| **ctHb**  **[g/dL)** | m | 11.8 ± 0.66 | 11.9 ± 0.44 | 12.1 ± 0.46 | 11.9 ± 0.44 | 12.1 ± 0.14 | 12.1 ± 0.14 | 12.2 ± 0.50 | 12.5 ± 0.44 |
|  | f | 12.0 ± 0.78 | 12.3 ± 0.67 | 12.1 ± 0.39 | 11.9 ± 0.41 | 12.6 ± 0.46 | 12.4 ± 0.32 | 12.4 ± 0.26 | 12.6 ± 0.41 |
| **sO_2_**  **[%]** | m | 86.8 ± 5.34 | 86.7 ± 4.94 | 86.7 ± 1.82 | 84.6 ± 3.15 | 87.2 ± 5.78 | 86.6 ± 4.89 | 86.7 ± 4.17 | 80.8 ± 4.94 |
|  | f | 86.4 ± 2.54 | 89.1 ± 2.84 | 86.6 ± 1.72 | 87.4 ± 2.73 | 85.8 ± 3.57 | 88.2 ± 2.28 | 87.1 ± 4.05 | 85.0 ± 3.79 |
| **Hctc**  **[%]** | m | 36.5 ± 1.98 | 36.5 ± 1.30 | 37.1 ± 1.60 | 36.8 ± 1.36 | 37.3 ± 0.69 | 37.3 ± 1.19 | 37.5 ± 1.54 | 38.4 ± 1.34 |
|  | f | 38.8 ± 2.40 | 37.8 ± 2.08 | 37.4 ± 1.23 | 36.7 ± 1.26 | 38.2 ± 0.88 | 39.0 ± 1.42 | 38.2 ± 0.71 | 38.9 ± 1.22 |
| **cK^+^**  **[mmol/L]** | m | **4.29 ± 0.13** | 4.54 ± 0.16 | 4.41 ± 0.12 | **4.63 ± 0.21** | 4.27 ± 0.24 | **4.71 ± 0.39** | 4.23 ± 0.21 | **4.65 ± 0.15** |
|  | f | **4.51 ± 0.19** | **4.18 ± 0.12** | 4.38 ± 0.14 | 4.35 ± 0.14 | 4.30 ± 0.27 | 4.40 ± 0.24 | 4.50 ± 0.15 | **4.19 ± 0.18** |
| **cNa^+^**  **[mmol/L]** | m | 136 ± 3.09 | 137 ± 2.52 | 136 ± 2.56 | 136 ± 2.23 | 138 ± 2.94 | 137 ± 3.44 | 136 ± 3.89 | 136 ± 0.90 |
|  | f | 136 ± 2.14 | 137 ± 1.60 | 136 ± 3.34 | 137 ± 2.88 | 136 ± 3.12 | 137 ± 2.62 | 136 ± 3.25 | 135 ± 2.33 |
| **cCa^2+^**  **[mmol/L]** | m | 1.16 ± 0.06 | 1.24 ± 0.07 | 1.24 ± 0.06 | 1.23 ± 0.08 | 1.26 ± 0.09 | 1.24 ± 0.08 | 1.19 ± 0.10 | 1.25 ± 0.09 |
|  | f | 1.26 ± 0.105 | 1.24 ± 0.04 | 1.23 ± 0.10 | 1.24 ± 0.09 | 1.21 ± 0.08 | 1.23 ± 0.05 | 1.21 ± 0.07 | 1.18 ± 0.09 |
| **cCl^-^**  **[mmol/L]** | m | 103 ± 2.24 | 103 ± 1.51 | 101 ± 1.21 | 104 ± 1.57 | 103 ± 0.69 | 103 ± 0.95 | 103 ± 1.31 | 101 ± 1.89 |
|  | f | 103 ± 1.00 | 103 ± 1.38 | 102 ± 1.41 | 104 ± 1.25 | 102 ± 1.64 | 102 ± 0.95 | 102 ± 1.85 | 102 ± 1.55 |
| **cHCO_3_^-^**  **[mmol/L]** | m | 25.1 ± 2.31 | 25.9 ± 1.94 | 26.7 ± 0.85 | 26.3 ± 1.98 | 26.5 ± 1.59 | 24.7 ± 2.89 | 24.9 ± 1.94 | 26.9 ± 2.33 |
|  | f | 26.9 ± 2.28 | 26.1 ± 0.66 | 25.9 ± 3.25 | 25.9 ± 2.00 | 25.4 ± 1.87 | 22.7 ± 5.00 | 25.3 ± 1.61 | 26.0 ± 2.91 |
| **ABEc**  **[mmol/L]** | m | 1.71 ± 1.64 | 2.30 ± 1.17 | 3.48 ± 1.54 | 1.69 ± 0.89 | 2.59 ± 0.99 | 1.49 ± 1.58 | 1.43 ± 1.41 | 2.83 ± 1.87 |
|  | f | 2.21 ± 1.87 | 1.74 ± 1.31 | 2.20 ± 1.84 | 1.80 ± 1.27 | 1.94 ± 1.41 | 1.70 ± 0.88 | 1.68 ± 1.63 | 2.39 ± 1.83 |
| **cGlu**  **[mmol/L]** | m | 8.08 ± 0.51 | 8.63 ± 0.57 | 8.50 ± 0.32 | 8.68 ± 0.27 | 7.91 ± 0.30 | 8.25 ± 0.75 | 8.00 ± 0.56 | 8.18 ± 0.86 |
|  | f | 8.10 ± 0.79 | 8.31 ± 0.49 | 8.65 ± 0.48 | 8.09 ± 0.57 | 7.90 ± 0.29 | 8.13 ± 0.52 | 7.73 ± 0.49 | 7.48 ± 0.41 |
| **cLac**  **[mmol/L]** | m | 2.25 ± 0.59 | 2.33 ± 0.61 | 1.51 ± 0.65 | 1.83 ± 0.60 | 2.41 ± 0.34 | 2.60 ± 0.37 | 2.56 ± 0.80 | 2.21 ± 1.10 |
|  | f | 2.20 ± 0.54 | 2.68 ± 0.13 | 2.53 ± 0.69 | 2.53 ± 0.54 | 2.68 ± 0.54 | 2.96 ± 0.48 | 2.53 ± 0.82 | 2.35 ± 1.25 |

This table represents the blood gas analysis at postnatal day 33-35 for male (m) and female (f) rats. Values are presented as mean ± standard deviation (SD) for the control diet and diets supplemented with lutein, docosahexaenoic acid (DHA), choline, or their combinations (n=6-9 per group and sex). Significant differences *versus* the sex-matched control group, as determined by Kruskal-Wallis Test followed by Dunn’s post-hoc tests for multiple comparisons, are indicated in bold.

**Supplementary Table 2:** **Clinical blood parameters at postnatal day 33-35.**

|  | | **Control** | **Lutein** | **DHA** | **Choline** | **Lutein**  **DHA** | **Lutein**  **Choline** | **DHA**  **Choline** | **Lutein**  **DHA**  **Choline** |
| --- | --- | --- | --- | --- | --- | --- | --- | --- | --- |
| **ALT [U/I]** | m | 31.4 ± 8.37 | 30.9 ± 6.31 | 33.0 ± 7.30 | 32.8 ± 7.07 | 30.1 ± 4.87 | 30.0 ± 5.67 | 32.9 ± 4.17 | 35.7 ± 6.29 |
|  | f | 28.8 ± 5.80 | 29.0 ± 4.76 | 29.5 ± 5.21 | 27.8 ± 4.68 | 27.9 ± 5.53 | 26.5 ± 2.84 | 29.4 ± 3.53 | 29.6 ± 5.71 |
| **AST [U/I]** | m | **69.4 ± 9.40** | 77.0 ± 10.2 | 75.9 ± 15.9 | **86.8 ± 9.34** | 78.7 ± 11.7 | 76.5 ± 10.6 | 80.1 ± 4.61 | 80.5 ± 9.87 |
|  | f | 78.9 ± 11.2 | 80.3 ± 19.0 | 76.1 ± 8.65 | 82.1 ± 11.6 | 72.8 ± 18.2 | 74.1 ± 7.62 | 78.9 ± 4.95 | 78.7 ± 7.46 |
| **Calcium_total_**  **[mmol/L]** | m | **2.46 ± 0.18** | 2.40 ± 0.41 | 2.38 ± 0.23 | 2.61 ± 0.18 | 2.50 ± 0.15 | 2.64 ± 0.06 | **2.69 ± 0.07** | 2.61 ± 0.14 |
|  | f | **2.63 ± 0.13** | 2.54 ± 0.16 | 2.43 ± 0.21 | 2.63 ± 0.12 | **2.26 ± 0.14** | 2.57 ± 0.05 | 2.63 ± 0.09 | 2.63 ± 0.13 |
| **Cholesterol**  **[mg/dL]** | m | 84.8 ± 11.0 | 83.9 ± 7.78 | 77.0 ± 8.78 | 95.1 ± 8.08 | 79.4 ± 9.51 | 101.0 ± 13.0 | 95.6 ± 13.8 | 91.6 ± 13.4 |
|  | f | 91.3 ± 9.67 | 86.2 ± 13.2 | 81.4 ± 7.33 | 98.2 ± 14.8 | 74.1 ± 14.4 | 96.4 ± 11.5 | 85.6 ± 19.7 | 81.9 ± 7.18 |
| **Crea**  **[mg/dL]** | m | 0.12 ± 0.04 | 0.13 ± 0.03 | 0.11 ± 0.03 | 0.13 ± 0.02 | 0.14 ± 0.04 | 0.15 ± 0.04 | 0.16 ± 0.06 | 0.13 ± 0.02 |
|  | f | 0.15 ± 0.03 | 0.15 ± 0.03 | 0.13 ± 0.03 | 0.14 ± 0.02 | 0.13 ± 0.02 | 0.14 ± 0.02 | 0.15 ± 0.02 | 0.16 ± 0.03 |
| **HS-CRP [mg/L]** | m | 0.10 ± 0.04 | 0.13 ± 0.05 | 0.08 ± 0.05 | 0.06 ± 0.02 | 0.13 ± 0.05 | 0.08 ± 0.04 | 0.07 ± 0.04 | 0.06 ± 0.04 |
|  | f | 0.09 ± 0.05 | 0.15 ± 0.02 | 0.06 ± 0.00 | 0.07 ± 0.04 | 0.13 ± 0.03 | 0.07 ± 0.05 | 0.05 ± 0.03 | 0.10 ± 0.05 |
| **HDL [mg/dL]** | m | **62.7 ± 9.29** | 63.6 ± 6.88 | 63.5 ± 3.57 | 74.3 ± 13.7 | 55.9 ± 7.00 | **76.8 ± 9.08** | 70.6 ± 13.1 | 62.6 ± 6.02 |
|  | f | 60.5 ± 12.4 | 61.7 ± 14.2 | 57.8 ± 6.31 | 69.7 ± 13.9 | 50.8 ± 8.64 | 67.3 ± 10.0 | 62.4 ± 15.5 | 57.6 ± 6.71 |
| **Sodium**  **[mmol/L]** | m | 132 ± 5.99 | 137 ± 7.62 | 133 ± 6.84 | 138 ± 4.14 | 138 ± 3.55 | 139 ± 2.12 | 139 ± 1.51 | 130 ± 2.69 |
|  | f | 133 ± 3.99 | 135 ± 6.31 | 133 ± 5.75 | 138 ± 4.20 | 128 ± 5.43 | 133 ± 4.66 | 138 ± 1.98 | 138 ± 2.00 |
| **Phos [mmol/L]** | m | 3.09 ± 0.12 | 2.99 ± 0.27 | 3.07 ± 0.27 | 3.06 ± 0.25 | 2.95 ± 0.22 | 3.08 ± 0.16 | 3.12 ± 0.21 | 3.14 ± 0.19 |
|  | f | **3.09 ± 0.17** | 2.97 ± 0.17 | 2.89 ± 0.06 | 2.97 ± 0.11 | **2.76 ± 0.22** | 2.86 ± 0.19 | 3.02 ± 0.19 | 3.08 ± 0.22 |
| **Protein_total_ [g/L]** | m | **45.2 ± 2.78** | 46.4 ± 5.08 | 47.5 ± 2.07 | 49.9 ± 2.85 | 47.1 ± 1.76 | **49.9 ± 1.67** | **51.4 ± 1.21** | **51.0 ± 1.50** |
|  | f | 50.8 ± 4.38 | 49.9 ± 3.45 | 48.1 ± 3.01 | 49.5 ± 2.87 | 47.7 ± 2.22 | 49.2 ± 2.24 | 50.3 ± 1.17 | 51.4 ± 2.81 |
| **Triglycerides [mg/dL]** | m | 99.1 ± 41.9 | 95.7 ± 47.3 | 87.5 ± 26.4 | 93.3 ± 32.5 | 82.5 ± 22.4 | 124 ± 37.1 | 103 ± 32.7 | 125 ± 51.6 |
|  | f | 116 ± 58.4 | 87.9 ± 31.2 | 97.7 ± 21.9 | 104 ± 60.1 | 89.4 ± 31.9 | 114 ± 40.2 | 110 ± 47.4 | 81.3 ± 19.0 |
| **Urea [mg/dL]** | m | 22.1 ± 5.28 | 24.7 ± 4.15 | 22.6 ± 3.98 | 23.1 ± 3.31 | 19.8 ± 1.73 | 23.5 ± 2.83 | 23.8 ± 5.99 | 24.7 ± 3.76 |
|  | f | 23.1 ± 3.79 | 25.3 ± 4.99 | 21.9 ± 3.52 | 21.4 ± 4.35 | 21.3 ± 5.48 | 24.0 ± 11.4 | 24.2 ± 4.85 | 22.0 ± 9.29 |

This table represents clinical blood parameters at postnatal day 33-35 for male (m) and female (f) rats. Values are presented as mean ± standard deviation (SD) for the control diet and diets supplemented with lutein, docosahexaenoic acid (DHA), choline, or their combinations (n=6-9 per group and sex). Significant differences *versus* the sex-matched control group, as determined by Kruskal-Wallis Test followed by Dunn’s post-hoc tests for multiple comparisons, are indicated in bold.

**Supplementary Table 3:** **Blood gas analysis at postnatal day 146-148.**

|  | | **Control** | **Lutein** | **DHA** | **Choline** | **Lutein**  **DHA** | **Lutein**  **Choline** | **DHA**  **Choline** | **Lutein**  **DHA**  **Choline** |
| --- | --- | --- | --- | --- | --- | --- | --- | --- | --- |
| **pH** | m | 7.42 ± 0.03 | 7.45 ± 0.04 | 7.43 ± 0.03 | 7.42 ± 0.04 | 7.44 ± 0.04 | 7.44 ± 0.04 | 7.46 ± 0.03 | 7.43 ± 0.02 |
|  | f | 7.41 ± 0.07 | 7.46 ± 0.07 | 7.42 ± 0.05 | 7.42 ± 0.04 | 7.43 ± 0.03 | 7.43 ± 0.03 | 7.42 ± 0.04 | **7.45 ± 0.05** |
| **pCO_2_**  **[mmHg]** | m | 37.0 ± 12.5 | 36.5 ± 5.06 | 40.5 ± 4.39 | 41.5 ± 7.22 | 40.0 ± 3.42 | 40.3 ± 5.03 | 38.3 ± 3.18 | 41.4 ± 3.38 |
|  | f | 41.1 ± 7.50 | 37.6 ± 7.21 | 40.3 ± 4.75 | 40.5 ± 4.22 | 40.1 ± 4.27 | 41.3 ± 4.85 | 42.4 ± 1.90 | 38.3 ± 6.11 |
| **ctHb**  **[g/dL)** | m | 15.6 ± 1.09 | 15.2 ± 0.20 | 15.6 ± 0.45 | 15.5 ± 0.62 | 14.8 ± 0.77 | 15.6 ± 0.61 | 15.3 ± 0.62 | 15.5 ± 0.61 |
|  | f | 15.7 ± 0.80 | 15.5 ± 0.68 | 15.5 ± 0.56 | 15.6 ± 0.42 | 15.4 ± 0.48 | 15.6 ± 0.45 | 15.4 ± 0.54 | 15.0 ± 0.66 |
| **sO_2_**  **[%]** | m | 90.5 ± 3.44 | 91.7 ± 4.17 | 90.1 ± 2.96 | 90.8 ± 3.76 | 87.2 ± 6.51 | 91.8 ± 3.10 | 93.5 ± 2.64 | 91.6 ± 3.59 |
|  | f | 91.5 ± 2.78 | 92.7 ± 3.99 | 90.8 ± 0.94 | 89.3 ± 2.70 | 94.1 ± 2.45 | 91.2 ± 3.95 | 92.4 ± 3.56 | 91.3 ± 2.10 |
| **Hctc**  **[%]** | m | 47.7 ± 3.24 | 46.2 ± 0.83 | 47.7 ± 1.38 | 47.4 ± 1.95 | 45.5 ± 2.30 | 44.2 ± 5.41 | 46.8 ± 1.93 | 47.4 ± 1.81 |
|  | f | 48.2 ± 2.44 | 47.5 ± 2.00 | 47.6 ± 1.67 | 47.9 ± 1.26 | 47.1 ± 1.38 | 47.7 ± 1.46 | 47.1 ± 1.60 | 47.3 ± 2.09 |
| **cK^+^**  **[mmol/L]** | m | 4.23 ± 0.27 | 4.20 ± 0.17 | 4.18 ± 0.20 | 4.20 ± 0.16 | 4.05 ± 0.14 | 4.30 ± 0.17 | 4.05 ± 0.12 | 4.34 ± 0.10 |
|  | f | 3.93 ± 0.21 | 3.84 ± 0.17 | 3.90 ± 0.13 | 3.99 ± 0.16 | 4.08 ± 0.23 | 4.16 ± 0.13 | 4.04 ± 0.16 | 3.97 ± 0.16 |
| **cNa^+^**  **[mmol/L]** | m | 140 ± 2.77 | 136 ± 6.09 | 140 ± 1.16 | 140 ± 1.13 | 138 ± 2.07 | 140 ± 1.07 | 139 ± 2.04 | 140 ± 0.75 |
|  | f | 139 ± 0.75 | 138 ± 1.98 | 138 ± 4.19 | 139 ± 1.06 | 139 ± 1.45 | 139 ± 0.58 | 139 ± 2.67 | 140 ± 0.76 |
| **cCa^2+^**  **[mmol/L]** | m | 1.24 ± 0.04 | 1.23 ± 0.06 | 1.28 ± 0.04 | 1.28 ± 0.07 | 1.23 ± 0.05 | 1.29 ± 0.05 | 1.21 ± 0.07 | 1.28 ± 0.04 |
|  | f | 1.23 ± 0.05 | 1.20 ± 0.10 | 1.21 ± 0.03 | 1.27 ± 0.04 | 1.25 ± 0.05 | 1.26 ± 0.03 | 1.24 ± 0.06 | 1.26 ± 0.06 |
| **cCl^-^**  **[mmol/L]** | m | 108 ± 1.57 | 107 ± 1.58 | 108 ± 1.28 | 107 ± 1.73 | 106 ± 2.14 | 106 ± 0.98 | 108 ± 1.27 | 106 ± 0.79 |
|  | f | 110 ± 1.76 | 108 ± 1.41 | 108 ± 0.92 | 109 ± 2.10 | 107 ± 2.00 | 108 ± 1.46 | 109 ± 2.83 | 108 ± 1.36 |
| **cHCO_3_^-^**  **[mmol/L]** | m | 25.8 ± 3.13 | 25.6 ± 0.91 | 26.1 ± 1.84 | 26.1 ± 2.05 | 26.7 ± 0.86 | 27.1 ± 1.51 | 26.9 ± 1.30 | 27.0 ± 1.28 |
|  | f | 25.5 ± 1.85 | 25.5 ± 1.72 | 25.0 ± 1.42 | 26.0 ± 1.60 | 26.2 ± 1.20 | 26.6 ± 1.70 | 26.0 ± 0.87 | 25.8 ± 1.86 |
| **ABEc**  **[mmol/L]** | m | 1.69 ± 1.95 | 1.87 ± 1.17 | 2.03 ± 1.36 | 1.96 ± 0.69 | 2.83 ± 1.12 | 3.23 ± 0.91 | 3.47 ± 1.05 | 2.84 ± 0.92 |
|  | f | 1.23 ± 1.88 | 1.94 ± 1.33 | 1.06 ± 1.58 | 2.44 ± 0.81 | 2.30 ± 0.72 | 2.40 ± 1.19 | 1.89 ± 0.91 | 2.28 ± 1.47 |
| **cGlu**  **[mmol/L]** | m | 9.00 ± 1.17 | 8.51 ± 2.02 | 9.41 ± 0.54 | 8.89 ± 0.62 | 9.50 ± 0.97 | 9.61 ± 0.56 | 9.16 ± 0.85 | 9.43 ± 0.72 |
|  | f | 8.98 ± 0.48 | 8.79 ± 0.72 | 8.50 ± 1.96 | 9.28 ± 0.83 | 9.33 ± 0.63 | 9.19 ± 0.45 | 9.29 ± 0.58 | 8.91 ± 0.61 |
| **cLac**  **[mmol/L]** | m | 1.96 ± 0.57 | 2.14 ± 0.80 | 2.03 ± 1.14 | 2.23 ± 0.78 | 1.82 ± 0.50 | 1.91 ± 0.71 | 1.83 ± 0.35 | 1.97 ± 0.49 |
|  | f | 1.82 ± 0.92 | 1.80 ± 0.62 | 1.81 ± 0.80 | 1.78 ± 0.83 | 1.69 ± 0.48 | 1.99 ± 0.47 | 1.89 ± 0.55 | 1.78 ± 0.62 |

This table represents the blood gas analysis at postnatal day 146-148 for male (m) and female (f) rats. Values are presented as mean ± standard deviation (SD) for the control diet and diets supplemented with lutein, docosahexaenoic acid (DHA), choline, or their combinations (n=6-9 per group and sex). Statistical analysis by Kruskal-Wallis Test followed by Dunn’s post-hoc tests for multiple comparisons revealed no significant differences.

**Supplementary Table 4:** **Clinical blood parameters at postnatal day 146-148.**

|  | | **Control** | **Lutein** | **DHA** | **Choline** | **Lutein**  **DHA** | **Lutein**  **Choline** | **DHA**  **Choline** | **Lutein**  **DHA**  **Choline** |
| --- | --- | --- | --- | --- | --- | --- | --- | --- | --- |
| **ALT [U/I]** | m | **28.2 ± 4.36** | 27.9 ± 6.00 | 28.8 ± 3.93 | 28.6 ± 4.65 | 19.7 ± 7.72 | **19.6 ± 6.10** | 18.5 ± 8.24 | 23.9 ± 3.29 |
|  | f | 28.4 ± 6.51 | 29.5 ± 6.47 | 24.3 ± 11.4 | 21.2 ± 5.20 | 26.3 ± 5.22 | 21.8 ± 4.53 | 20.7 ± 8.58 | 25.9 ± 5.19 |
| **AST [U/I]** | m | 64.5 ± 8.78 | 64.4 ± 12.2 | 74.8 ± 17.3 | 71.5 ± 18.2 | 53.4 ± 17.2 | 49.0 ± 9.33 | 55.0 ± 7.28 | 51.3 ± 16.6 |
|  | f | 60.3 ± 13.8 | 81.0 ± 23.4 | 66.0 ± 14.4 | 55.4 ± 13.3 | 59.5 ± 16.0 | 43.3 ± 16.2 | 45.3 ± 11.6 | 60.2 ± 16.7 |
| **Calcium_total_**  **[mmol/L]** | m | **2.76 ± 0.11** | 2.49 ± 0.24 | **2.22 ± 0.25** | 2.40 ± 0.23 | **2.04 ± 0.48** | **2.08 ± 0.30** | **1.89 ± 0.55** | **2.29 ± 0.20** |
|  | f | 2.27 ± 0.24 | 2.41 ± 0.13 | 2.40 ± 0.14 | 1.96 ± 0.13 | 2.26 ± 0.21 | 1.98 ± 0.29 | 1.88 ± 0.11 | 2.43 ± 0.25 |
| **Cholesterol**  **[mg/dL]** | m | 104 ± 11.2 | 110 ± 26.3 | 101 ± 10.2 | 102 ± 12.5 | 76.7 ± 26.0 | 81.7 ± 16.1 | 95.8 ± 24.4 | 99.6 ± 27.0 |
|  | f | 81.0 ± 10.4 | 82.8 ± 19.7 | 82.9 ± 12.4 | 71.0 ± 11.7 | 84.2 ± 14.9 | 65.9 ± 13.7 | 62.8 ± 18.0 | 72.1 ± 5.32 |
| **Crea**  **[mg/dL]** | m | 0.27 ± 0.02 | 0.30 ± 0.03 | 0.29 ± 0.05 | 0.24 ± 0.05 | 0.24 ± 0.03 | 0.26 ± 0.02 | 0.29 ± 0.03 | 0.26 ± 0.02 |
|  | f | 0.31 ± 0.05 | 0.30 ± 0.03 | 0.29 ± 0.05 | 0.25 ± 0.02 | 0.28 ± 0.03 | 0.29 ± 0.02 | 0.25 ± 0.09 | 0.29 ± 0.03 |
| **HS-CRP [mg/L]** | m | 0.13 ± 0.05 | 0.15 ± 0.04 | 0.14 ± 0.04 | 0.14 ± 0.05 | 0.13 ± 0.07 | 0.12 ± 0.03 | 0.14 ± 0.02 | 0.15 ± 0.04 |
|  | f | 0.14 ± 0.03 | 0.14 ± 0.04 | 0.14 ± 0.04 | 0.13 ± 0.03 | 0.12 ± 0.02 | 0.12 ± 0.04 | 0.13 ± 0.04 | 0.15 ± 0.05 |
| **HDL [mg/dL]** | m | 57.2 ± 5.35 | 63.4 ± 17.6 | 52.3 ± 6.31 | 73.1 ± 13.4 | 42.1 ± 9.95 | 41.0 ± 8.66 | 49.3 ± 12.8 | 54.5 ± 19.2 |
|  | f | 50.2 ± 9.86 | 58.8 ± 15.2 | 53.6 ± 7.33 | 48.6 ± 10.2 | 50.3 ± 9.04 | 45.1 ± 9.78 | 42.8 ± 11.3 | 48.6 ± 11.2 |
| **Sodium**  **[mmol/L]** | m | 135 ± 4.63 | 137 ± 4.47 | 129 ± 7.09 | 128 ± 4.20 | 130 ± 3.85 | 126 ± 5.47 | 118 ± 16.90 | 131 ± 7.10 |
|  | f | 130 ± 7.31 | 137 ± 1.95 | 136 ± 5.01 | 125 ± 4.77 | 132 ± 5.55 | 130 ± 6.57 | 124 ± 5.50 | 131 ± 4.53 |
| **Phos [mmol/L]** | m | 1.86 ± 0.17 | 1.96 ± 0.12 | 1.92 ± 0.24 | 1.90 ± 0.06 | 1.74 ± 0.10 | 1.68 ± 0.15 | 1.77 ± 0.21 | 1.75 ± 0.12 |
|  | f | 1.67 ± 0.20 | 1.69 ± 0.11 | 1.80 ± 0.15 | 1.55 ± 0.24 | 1.74 ± 0.12 | 1.88 ± 0.53 | 1.84 ± 0.23 | 1.66 ± 0.07 |
| **Protein_total_ [g/L]** | m | **57.3 ± 3.71** | 59.7 ± 1.77 | 55.5 ± 4.18 | 56.3 ± 2.26 | 47.4 ± 8.39 | **45.6 ± 4.87** | 52.6 ± 5.31 | 46.2 ± 15.7 |
|  | f | 57.2 ± 4.25 | 59.2 ± 3.31 | 58.2 ± 4.31 | 52.6 ± 4.82 | 55.1 ± 5.38 | 49.2 ± 4.99 | 50.50± 10.7 | 53.9 ± 2.9 |
| **Triglycerides [mg/dL]** | m | 174 ± 53.8 | 168 ± 83.4 | 169 ± 55.6 | 171 ± 66.7 | 147 ± 98.0 | 139 ± 26.1 | 177 ± 86.7 | 245 ± 208 |
|  | f | 77.8 ± 26.4 | 81.2 ± 28.6 | 79.1 ± 27.5 | 73.3 ± 35.4 | 83.4 ± 17.9 | 121 ± 68.6 | 83.1 ± 42.1 | 74.3 ± 32.9 |
| **Urea [mg/dL]** | m | 27.5 ± 3.74 | 30.9 ± 3.12 | 27.6 ± 2.11 | 29.0 ± 2.57 | 25.5 ± 2.39 | 26.7 ± 3.13 | 28.9 ± 4.33 | 27.4 ± 3.42 |
|  | f | 29.4 ± 3.86 | 28.3 ± 3.51 | 27.3 ± 1.57 | 26.8 ± 3.14 | 26.7 ± 1.74 | 32.1 ± 4.00 | 29.3 ± 4.30 | 29.1 ± 6.05 |

This table represents clinical blood parameters at postnatal day 146-148 for male (m) and female (f) rats. Values are presented as mean ± standard deviation (SD) for the control diet and diets supplemented with lutein, docosahexaenoic acid (DHA), choline, or their combinations (n=6-9 per group and sex). Significant differences *versus* the sex-matched control group, as determined by Kruskal-Wallis Test followed by Dunn’s post-hoc tests for multiple comparisons, are indicated in bold.

**Supplementary Table 5: Nutrient composition of basal Altromin C1000 diet.**

| **Ingredient** | **Unit** | **Content** |
| --- | --- | --- |
| Crude Protein | mg/kg | 176115.000 |
| Crude Fat | mg/kg | 50830.000 |
| Crude Fibre | mg/kg | 40450.000 |
| Crude Ash | mg/kg | 54943.225 |
| Moisture | mg/kg | 81735.625 |
| Disaccharide(s) | mg/kg | 110960.500 |
| Polysaccharide(s) | mg/kg | 471700.000 |
| Metab. Energy | kcal/kg | 3518.055 |
| Lysine | mg/kg | 17400.970 |
| Methionine | mg/kg | 10688.000 |
| Cystine | mg/kg | 3196.180 |
| Threonine | mg/kg | 7154.170 |
| Tryptophan | mg/kg | 1976.960 |
| Arginine | mg/kg | 9828.790 |
| Histidine | mg/kg | 5275.790 |
| Isoleucine | mg/kg | 7222.820 |
| Leucine | mg/kg | 14762.770 |
| Phenylalanine | mg/kg | 7171.970 |
| Valine | mg/kg | 3296.140 |
| Alanine | mg/kg | 2528.000 |
| Aspartic acid | mg/kg | 3583.140 |
| Glutamic acid | mg/kg | 23674.970 |
| Glycine | mg/kg | 3136.000 |
| Proline | mg/kg | 12762.980 |
| Serine | mg/kg | 5267.800 |
| Tyrosine | mg/kg | 9285.010 |
| Vitamin A | I.E./kg | 15000.000 |
| Vitamin D3 | I.E./kg | 500.000 |
| Vitamin E | mg/kg | 163.900 |
| Vitamin K3 as Menadione | mg/kg | 10.000 |
| Vitamin B1 | mg/kg | 20.040 |
| Vitamin B2 | mg/kg | 20.322 |
| Vitamin B6 | mg/kg | 15.034 |
| Vitamin B12 | mg/kg | 0.030 |
| Nicotinic acid | mg/kg | 50.170 |
| Pantothenic acid | mg/kg | 50.106 |
| Folic acid | mg/kg | 10.00240 |
| Biotin | mg/kg | 0.201 |
| Choline chloride | mg/kg | 1011.500 |
| Benzoic acid | mg/kg | 100.000 |
| Inositol | mg/kg | 111.000 |
| Vitamin C | mg/kg | 20.000 |
| Calcium | mg/kg | 9310.506 |
| Phosphorus | mg/kg | 7522.765 |
| Digest.Phosporus | mg/kg | 7199.565 |
| Magnesium | mg/kg | 683.506 |
| Sodium | mg/kg | 2488.262 |
| Potassium | mg/kg | 7088.682 |
| Sulfur | mg/kg | 2791.540 |
| Chlorine | mg/kg | 3630.000 |
| Iron | mg/kg | 178.579 |
| Manganese | mg/kg | 100.888 |
| Zinc | mg/kg | 29.299 |
| Copper | mg/kg | 5.751 |
| Iodine | mg/kg | 0.514 |
| Molybdenum | mg/kg | 0.198 |
| Fluorine | mg/kg | 4.170 |
| Selenium | mg/kg | 0.334 |
| Cobalt | mg/kg | 0.147 |
| Capric acid C-10:0 | mg/kg | 2.500 |
| Lauric acid C-12:0 | mg/kg | 2.500 |
| Myristic acid C-14:0 | mg/kg | 2.500 |
| Pentadecanoic acid C-15:0 | mg/kg | 2.500 |
| Palmitic acid C-16:0 | mg/kg | 2700.000 |
| Palmitoleic acid C-16:1 | mg/kg | 2.500 |
| Margaric acid | mg/kg | 2.500 |
| Stearic acid C-18:0 | mg/kg | 1250.000 |
| Oleic acid C-18:1 | mg/kg | 10950.000 |
| Linoleic acid C-18:2 | mg/kg | 35050.000 |
| Alpha-linolenic acid C-18:3 | mg/kg | 150.000 |
| Arachidic acid C-20:0 | mg/kg | 250.000 |
| Eicosanoic acid C-20:1 | mg/kg | 250.000 |
| Eicosadienoic acid C-20:2 | mg/kg | 250.000 |
| Arachidonic acid C-20:4 | mg/kg | 2.500 |
| Eicosapentaenoic acid C-20:5 | mg/kg | 2.500 |
| Behenic acid C-22:0 | mg/kg | 250.000 |
| Docosahexaenoic acid C-22:6 | mg/kg | 2.500 |
| Tricosanoic acid | mg/kg | 2.500 |
| Nervonic acid C-24:1 | mg/kg | 2.500 |
| Erucic acid C-22:1 | mg/kg | 2.500 |
| Aluminium | mg/kg | 3.706 |
| Volume | kg | Σ 1000.000 |

This table lists all ingredients of the basal Altromin C1000 diet provided to all animals during the study, along with their respective quantities or concentrations.
